# Strength Test and Mechanism Research of Nano Calcium Carbonate–Cement Solidified Dredged Sludge

**DOI:** 10.3390/ma19091787

**Published:** 2026-04-28

**Authors:** Qizhi Hu, Ke Zhang, Qiang Ma, Gaoliang Tao

**Affiliations:** 1 School of Civil Engineering, Architecture and Environment, Hubei University of Technology, Wuhan 430068, China; hqz0716@163.com (Q.H.); maqiang927@163.com (Q.M.); tgl1979@126.com (G.T.); 2 Bridge Safety Monitoring Technology and Equipment Engineering Technology Research Center, Hubei University of Technology, Wuhan 430068, China

**Keywords:** dredged sludge, cement, nano-calcium carbonate, unconfined compressive strength, microscopic mechanism

## Abstract

**Highlights:**

- A composite binder of 8% cement and 2% nano-CaCO_3_ (by cement mass) increases the 28-day unconfined compressive strength of dredged sludge from 213 kPa to 721 kPa, representing a 21% improvement over cement-only stabilization.
- The addition of nano-CaCO_3_ accelerates early-age hydration, promotes C-S-H gel formation, and converts less stable AFm-SO_4_ into AFm-CO_3_, leading to a denser and more refined pore structure.
- The solidification mechanism combines three synergistic effects: nucleation (providing additional hydration sites), physical pore filling, and chemical reaction to form more stable phases.
- The findings offer a theoretical basis for designing low-carbon composite stabilizers and support the resource utilization of dredged sludge in subgrade applications.

**Abstract:**

This study investigates the valorization of dredged sludge as a sustainable subgrade fill material through stabilization with a nano-calcium carbonate–cement composite. Unconfined compressive strength (UCS) tests were systematically conducted to determine the optimal dosage of nano-CaCO_3_ as a supplementary additive at a fixed cement content of 8% by dry soil mass. Scanning electron microscopy (SEM), X-ray diffraction (XRD), and quantitative pore structure analysis were employed to elucidate the underlying solidification mechanisms. The results demonstrate that the addition of 2% nano-CaCO_3_ yields the highest 28-day UCS of 721 kPa, representing a statistically significant 21% improvement over the cement-only reference (596 kPa) and a more than threefold increase relative to untreated sludge (213 kPa). Conversely, increasing the nano-CaCO_3_ dosage to 2.5% leads to a significant strength reduction, attributed to nanoparticle agglomeration and hindered cement hydration. Microstructural characterization reveals that the optimal nano-CaCO_3_ dosage accelerates early-age hydration through a nucleation effect, promotes the consumption of portlandite, and enhances the formation of calcium silicate hydrate (C–S–H) gel. Semi-quantitative XRD analysis further confirms the conversion of less stable monosulfate (AFm-SO_4_) into stable monocarboaluminate (AFm-CO_3_) phases. These synergistic mechanisms—nucleation, physical pore filling, and chemical reaction—result in a densified matrix with a refined pore structure, reduced total porosity, and a more homogeneous pore-size distribution. The findings provide a robust theoretical basis for the resource-oriented utilization of dredged sludge and the design of low-carbon composite stabilizers for soft soil treatment.

## 1. Introduction

Dredging and channel maintenance activities generate large amounts of dredged sludge every year. Owing to its high water content, low strength, and poor engineering properties, the large-scale treatment of dredged sludge in China still mainly relies on disposal and simple landfilling [1,2]. With the implementation of national policies promoting the harmless treatment and resource utilization of sludge, such as the Implementation Plan for the Harmless Treatment and Resource Utilization of Sludge (Fagai Huanzi [2022] No. 1453), the beneficial reuse of dredged sludge has received increasing attention in recent years [3]. Among the available treatment methods, solidification/stabilization is considered one of the most effective approaches for converting dredged sludge into reusable geomaterials. Compared with untreated sludge, cement-solidified dredged sludge generally exhibits reduced pore volume and compressibility, together with improved compression modulus and shear strength [4]. Liu et al. [5] reported that the cohesion of cement-solidified dredged sludge increased exponentially with curing age and linearly with cement content. However, the extensive use of cement in sludge treatment is associated with high energy consumption, significant carbon emissions, and increased material cost. Therefore, alternative binders and supplementary modifiers have been widely investigated to improve treatment efficiency and reduce cement consumption [6,7,8].

With the rapid development of nanotechnology, nanomaterials have emerged as promising additives for cement-based materials. Nanomaterials are generally defined as ultrafine materials with particle sizes ranging from 1 to 100 nm [9,10]. Among them, nano-calcium carbonate (NC) has attracted considerable interest because of its high physicochemical activity, low cost, and good compatibility with cementitious systems [11]. Previous studies have shown that NC can accelerate cement hydration and improve the early-age mechanical performance of cement-based materials. Kim G et al. [12] found that NC promoted early hydration and increased the flexural and compressive strengths of hardened cement paste. Fu Q et al. [13] demonstrated that, in cement systems containing supplementary cementitious materials, increasing the NC dosage enhanced early hydration, mainly due to the seed crystal effect of NC and its promotion of C-S-H nucleation. In addition, many studies have confirmed that nanomaterials can improve the mechanical properties and durability of concrete [14,15,16]. Meng T et al. [17] reported that both NC and nano-silica enhanced the compressive and flexural strengths of concrete, while nano-silica also reduced pore volume and accelerated hydration through the pozzolanic effect. Zhang C et al. [18] found that a small amount of NC effectively improved the early compressive strength of mortar and concrete with high fly ash content. Zhuang et al. [19] further showed that nanomaterials improved the uniformity, compactability, and compressive strength of cement-stabilized soil by accelerating pozzolanic reactions and promoting the formation of C-S-H gel.

Despite these advances, previous studies on nanomaterials have mainly focused on concrete, mortar, and cement paste, whereas their application in cement-stabilized soils, especially dredged sludge, remains limited. This gap is significant because dredged sludge differs fundamentally from conventional cement-based materials. First, its initial water content is usually very high, often exceeding 90%, which dilutes the binder system and alters hydration kinetics. Second, dredged sludge may contain clay minerals and organic matter, both of which can interfere with cement hydration, particle agglomeration, and nanoparticle dispersion. Third, the target strength of stabilized dredged sludge is generally much lower than that of structural concrete and is typically in the range of 200–1000 kPa for subgrade-related applications. Therefore, the mechanisms by which nanomaterials improve concrete or cement paste cannot be directly extrapolated to dredged sludge systems.

In practical dredged sludge treatment, cement remains the primary binder. However, its large-scale use not only increases treatment cost but also raises environmental concerns. Against this background, the incorporation of nano-calcium carbonate into cement-stabilized dredged sludge deserves systematic investigation. In this study, nano-calcium carbonate was introduced as an additive into a cement-based stabilization system to evaluate the effects of different dosages on the strength development of dredged sludge. Unconfined compressive strength (UCS) tests were performed to identify the dosage range favorable for mechanical improvement, and the corresponding solidification mechanisms were investigated using scanning electron microscopy (SEM), X-ray diffraction (XRD), and the Particle (Pore) and Crack Analysis System (PCAS). These techniques were used to characterize hydration products, microstructural evolution, and pore-structure variation in the stabilized sludge.

The novelty of this study is twofold. First, it systematically investigates the role of nano-calcium carbonate in a compositionally complex dredged sludge matrix rather than in conventional cement-based materials. Second, it elucidates the coupled effects of nucleation, filling, and chemical interaction induced by NC through an integrated analysis of macroscopic strength behavior and microscopic structural evidence. The results are expected to provide a useful theoretical and experimental basis for the stabilization and resource utilization of dredged sludge using nanomaterial-modified binders.

## 2. Materials and Methods

### 2.1. Test Materials

**Figure 1:**
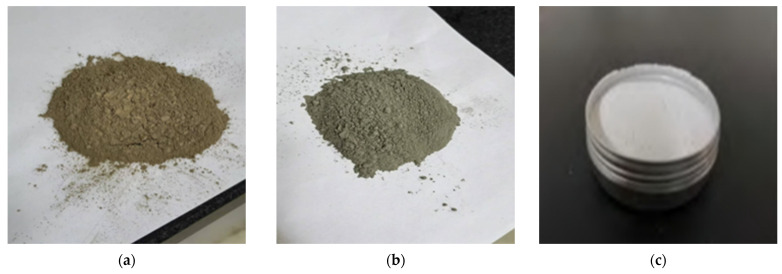
Test materials: (**a**) dredged sludge; (**b**) Portland cement; (**c**) nano-calcium carbonate.

The experiment utilized dredged sludge as the base material, with cement and nano-calcium carbonate serving as solidification agents, as shown in Figure 1. Representative test materials are shown in Table 1.

The raw sludge used in the experiment was collected from the riverbed between chainages K38 + 000 and K38 + 200 of the third contract section of the Bahe–Qizhou Highway (National Highway G347) in Huanggang City. The dredged sludge was in a saturated state, appearing dark gray with patches of black. It exhibited a smooth surface, good plasticity, and a pronounced fishy odor. The strong fishy odor indicates the presence of organic matter within the sludge. Such organic components, particularly humic acids, are known to potentially affect cement hydration reactions by adsorbing onto cement grain surfaces and neutralizing the alkaline pore solution, thereby retarding the formation of hydration products. Although the organic content was not quantified in the present study, this possible effect should be acknowledged when interpreting the results. Future work should include additional characterization such as total organic carbon (TOC) or loss-on-ignition (LOI) testing.

The basic physical properties determined through laboratory tests are presented in Table 1. The chemical composition of the dredged sludge is summarized in Table 2. Prior to use, the material was oven-dried, ground, and sieved through a 2 mm mesh. The test employed Grade 42.5 Portland cement as the cementitious material, manufactured by Huaxin Cement Co., Ltd., Wuhan, China. The chemical composition of the cement is summarized in Table 3, while the properties of the nano-calcium carbonate are detailed in Table 4. The nano-calcium carbonate was supplied by Hangzhou Hengge Nanotechnology Co., Ltd., Hangzhou, China, with an average particle size of 10–20 nm and purity > 99.9%.

### 2.2. Test Scheme

The dosage of nano-calcium carbonate is expressed as a percentage of the cement mass and is added as a supplementary admixture rather than as a cement replacement, whereas the dosages of cement and water are expressed as percentages of the dry soil mass. Previous studies have shown that increasing the cement content in cement-stabilized soil does not always lead to improved performance; in general, the economically optimal dosage is approximately 5%. For the specific application of nano-calcium carbonate–cement composite solidification of dredged sludge, excessively high cement content (reaching or exceeding 10%) would not only diminish practical applicability but also conflict with the principles of environmental sustainability. Therefore, the cement content was fixed at 8%, and the nano-calcium carbonate content was set to 1%, 1.5%, 2%, and 2.5% by mass of cement, with a water-to-cement ratio of 0.45. After curing to the specified ages, unconfined compressive strength tests were conducted (Table 5). The mixture exhibiting the optimal solidification performance was selected for further microstructural analysis using X-ray diffraction (XRD), scanning electron microscopy (SEM), and the Particle (Pore) and Crack Image Recognition and Analysis System (PCAS). The rationale for adding nano-CaCO_3_ as a supplementary admixture rather than a direct cement substitute is twofold: (i) a minimum cement content (approximately 8%) is required in dredged sludge stabilization to initiate sufficient hydration and provide basal strength; (ii) nano-CaCO_3_ functions effectively as a performance enhancer at low dosages, improving the efficiency of the existing cement without replacing its binding role.

### 2.3. Specimen Preparation

A specified amount of silt was taken and mixed with the appropriate proportions of cement and nano-calcium carbonate according to the test protocol. The mixture was placed into a mixer, and water was added at the specified ratio. Mixing was continued until the mixture was thoroughly homogenized. The test specimens were prepared by compacting the mixture in four layers using a three-part mold with a diameter of 39.1 mm and a height of 80 mm. The specimen dimensions, with a height-to-diameter ratio of approximately 2:1, were selected to minimize end-plate friction effects during uniaxial compression. Furthermore, the use of smaller-scale specimens facilitates the homogeneous dispersion of limited quantities of nanomaterials and allows for precise sampling of representative microstructural features for subsequent SEM and XRD analyses. Each layer was compacted 27 times, and a diamond-shaped scraper was applied between layers to enhance interlayer bonding. The surface of each specimen was finally leveled with a trowel. After preparation, the specimens, together with the molds, were placed in a standard constant-temperature curing chamber at 20 ± 2 °C. After 24 h, once the cement had achieved initial set, the specimens were demolded, wrapped in plastic wrap, and returned to the curing chamber until the designated curing age was reached.

### 2.4. Mechanical Testing and Statistical Analysis

Unconfined compressive strength (UCS) tests were conducted in accordance with ASTM D2166/D2166M-24 [20]. A computer-controlled universal testing machine was employed with a constant axial strain rate of 1.0 mm/min. Load and deformation data were recorded continuously until either a distinct peak load was observed or an axial strain of 15% was reached. For each mixture composition and curing age (7, 14, and 28 days), a minimum of three replicate specimens were prepared and tested. The reported UCS values represent the arithmetic mean of the three replicates. Any individual replicate deviating by more than 15% from the median value was considered an outlier and excluded from the final average; in such cases, an additional replicate was tested to maintain *n* = 3. Standard deviations were calculated and are reported as error bars in figures and as ± values in tables. Statistical significance of the UCS differences among mixture groups was evaluated using one-way analysis of variance (ANOVA) followed by Tukey’s post hoc test at a significance level of α = 0.05. The results of this analysis are presented in Section 3.1.

## 3. Results and Discussion

### 3.1. Unconfined Compressive Strength Test and Results Analysis

#### 3.1.1. Unconfined Compressive Strength Analysis of Stabilized Soil

**Figure 2:**
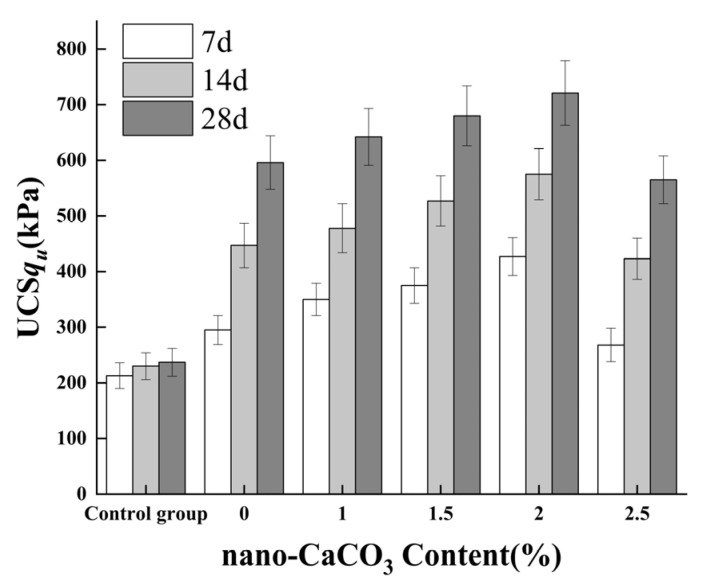
Unconfined Compressive Strength of Cured Soil at Different Ages with Varying Levels of Nano-Calcium Carbonate Addition. Error bars represent standard deviation (*n* = 3).

As shown in Figure 2, the addition of nano-calcium carbonate–cement significantly enhances the unconfined compressive strength of the stabilized dredged sludge. The compressive strength increases with curing age and exhibits a trend of first increasing and then decreasing with higher nano-calcium carbonate content. Across different curing ages, the unconfined compressive strength (q_u_) of the untreated dredged sludge was merely 213 kPa to 237 kPa, suggesting that curing age has no substantial effect on the strength of the raw sludge. Following cement addition, q_u_ of the cement-stabilized soil ranged from 295 kPa to 596 kPa, indicating that the strength contribution of cement becomes evident only after 7 days, with limited effect on early-stage strength.

When the nano-calcium carbonate content was increased from 1% to 2%, the strength of the stabilized soil improved across all curing ages. For specimens containing 2% nano-calcium carbonate, q_u_ ranged from 427 kPa to 721 kPa, corresponding to increases of 100%, 150%, and 204% at 7, 14, and 28 days, respectively, compared with the control group. Relative to the cement-stabilized group without nano-calcium carbonate, the compressive strengths at the same ages increased by 45%, 29%, and 21%, respectively. In contrast, at a dosage of 2.5%, a significant decrease in load-bearing capacity was observed. This phenomenon is primarily attributed to nanoparticle agglomeration. At excessive concentrations, the high surface energy of NCC particles induces flocculation and the formation of micro-sized agglomerates. These agglomerates act as localized weak zones or stress concentration points rather than effective nano-fillers. Additionally, the high specific surface area of the agglomerated nanoparticles may absorb a portion of the mixing water required for complete cement hydration, leading to a dilution effect on the continuity of the C-S-H gel matrix. These results indicate that the strength enhancement provided by nano-calcium carbonate–cement is most pronounced during the early curing stage (before 14 days), and its effectiveness gradually diminishes with prolonged curing.

Based on the 28-day unconfined compressive strength (q_u_) of the stabilized soil as the reference, the strength ratios at different curing ages are presented in Table 6. The unconfined compressive strengths at 7 days and 14 days exhibit a clear relationship with that at 28 days: the 7-day strength ranges from 47% to 59% of the 28-day strength, while the 14-day strength ranges from 74% to 80% of the 28-day strength, respectively.

**Figure 3:**
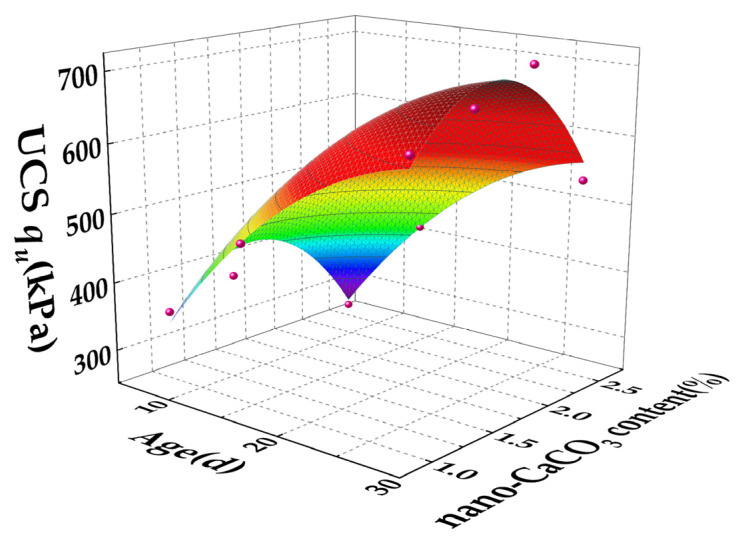
Effect of Nano-Calcium Carbonate Dosage and Curing Age on Strength of Cement-Stabilized Soil.

The compressive strength of nano-calcium carbonate–cement stabilized soil can be expressed as a function of the two main influencing factors—nano-calcium carbonate content and curing age—via regression analysis. The resulting unconfined compressive strengths for different dosages are shown in Figure 3.

A one-way ANOVA was conducted to assess the statistical significance of nano-CaCO_3_ dosage on the 28-day UCS. The analysis confirmed that nano-CaCO_3_ dosage has a statistically significant effect on strength development (*p* < 0.001). Tukey’s post hoc comparisons (Table 7) revealed that:

The 2% NCC mixture exhibited significantly higher strength than all other mixtures (*p* < 0.05), confirming the identification of 2% as the optimum dosage within the tested range.

The 1.5% and 2.5% mixtures did not differ significantly from each other (*p* > 0.05), although both were significantly higher than the 0% control.

The 2.5% mixture showed a significant reduction compared to the 2% mixture (*p* = 0.003), statistically validating the detrimental effect of overdosage.

#### 3.1.2. Analysis of the Failure Characteristics of Stabilized Soil

Cement-stabilized soil is ideally characterized by brittle failure, which signifies the formation of strong cementitious bonds between soil particles. Ductile or plastic behavior in stabilized soils generally indicates insufficient binder content or incomplete hydration, resulting in a matrix that deforms rather than fractures under load.

**Figure 4:**
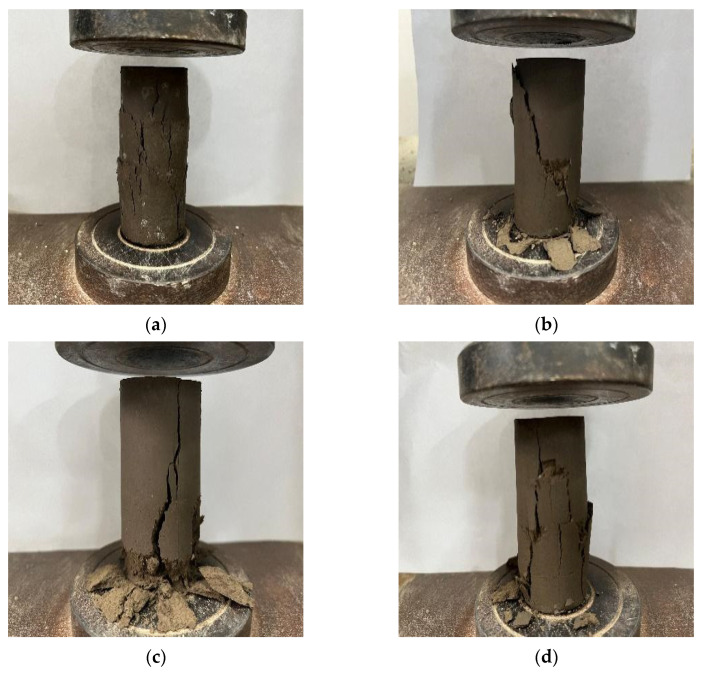
Comparison of Failure Characteristics in Typical Consolidated Soil Specimens. (**a**) Failure pattern of untreated sludge; (**b**) Failure pattern of cement-stabilized soil; (**c**) Failure pattern of 2% NC–cement-stabilized soil; (**d**) Failure pattern of 2.5% NC–cement-stabilized soil.

Figure 4a–d illustrate the failure modes of untreated silt, cement-stabilized soil, and nano-calcium carbonate–cement-stabilized soil after 28 days of curing.

Under uniaxial compression, the untreated silt initially undergoes compression (Figure 4a). As loading continues, cracks develop on the specimen surface, primarily concentrated in the central region, with no clear shear plane. Ultimately, the outer layer spalls and the soil mass fails, exhibiting overall plastic failure.

At a low nano-calcium carbonate content (Figure 4b), a distinct main crack forms in the stabilized soil upon failure. The fracture surface is inclined at a certain angle relative to the direction of vertical loading, consistent with the failure characteristics of cement-stabilized soil [21], indicating brittle shear failure.

At the optimal nano-calcium carbonate dosage (Figure 4c), fine cracks appear at the base of the composite-stabilized specimen, with the main crack propagating vertically, characteristic of brittle splitting failure.

In addition, a special failure mode was observed during testing, in which the specimen developed multiple intersecting cracks after failure, exhibiting features of both brittle and plastic failure (Figure 4d). This behavior is likely attributable to agglomeration caused by uneven dispersion of the nanoparticles within the soil matrix.

**Figure 5:**
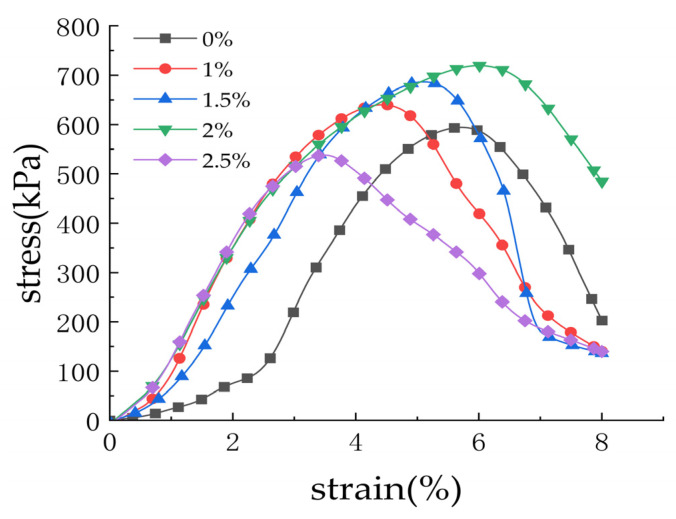
Stress–strain curves of cement-stabilized soil with different nano-calcium carbonate content.

As shown in Figure 5, the stress–strain curve indicates that with increasing nano-calcium carbonate content, the curve approximates a straight line when the axial strain is within 3%. During this stage, the slope is steep and the stress increases rapidly, suggesting that the composite stabilized soil undergoes strength failure in the initial stage of straining. From an axial strain of 3% up to the peak stress, the axial stress increases gradually, after which it begins to decline. The overall stress–strain curve of the stabilized soil follows a parabolic shape. The variation patterns of the stress–strain curves for cement-stabilized soil specimens before and after the addition of nano-calcium carbonate are essentially consistent, aligning with the failure modes described above. This indicates that the inclusion of nano-calcium carbonate does not alter the failure mode of cement-stabilized soil.

### 3.2. X-Ray Diffraction Test (XRD)

**Figure 6:**
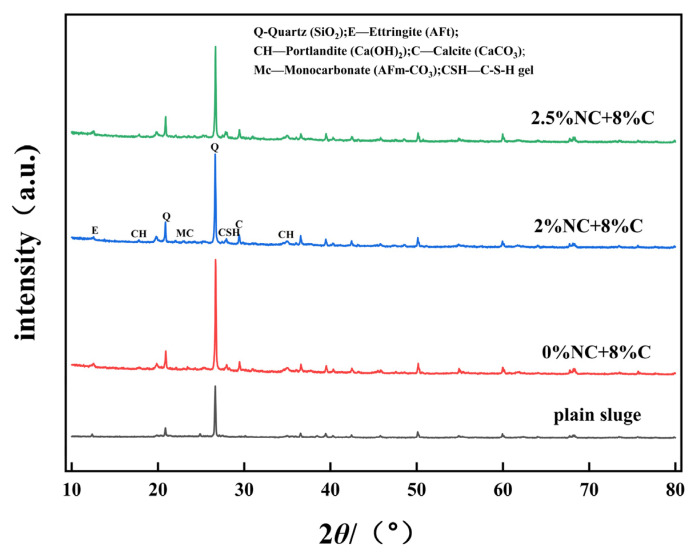
XRD spectrum of stabilized soil.

Representative specimens with a curing age of 28 days were selected for X-ray diffraction analysis. The XRD patterns of plain sludge, cement-stabilized soil (0%NC), and composite-stabilized soils with 2% and 2.5% nano-CaCO_3_ are presented in Figure 6. The primary hydration products generated during cement hydration include calcium silicate hydrate (C-S-H) gel, portlandite (Ca(OH)_2_) crystals, and ettringite (AFt) [22]. Among these, the enhancement of mechanical strength in cement-stabilized soil is mainly attributed to the formation of C-S-H gel and AFt crystals, whereas portlandite contributes minimally to strength development [23]. Furthermore, if tricalcium aluminate (C_3_A) remains after the complete consumption of gypsum, it may further react with ettringite to form AFm phases such as monosulfate (AFm-SO_4_) [24].

The stacked XRD patterns in Figure 6 reveal distinct differences in the crystalline phase assemblages among the four sample groups. The plain sludge exhibits strong quartz peaks (2θ ≈ 20.9° and 26.6°) and weak reflections from clay minerals, with no detectable cement hydration products. Upon cement addition (0%NC), characteristic peaks of portlandite (2θ ≈ 18.1°and 34.1°), ettringite (2θ ≈ 12.5°), and calcite (2θ ≈ 29.4°) emerge, confirming the occurrence of cement hydration. With the incorporation of 2% nano-CaCO_3_, notable changes are observed: the intensity of the portlandite peak decreases, while the calcite peak intensity increases substantially relative to the 0%NC mixture. Additionally, a slight reduction in the ettringite peak is observed, accompanied by changes in the AFm-CO_3_ region (2θ ≈ 23.1°). These trends suggest that nano-CaCO_3_ promotes further consumption of Ca(OH)_2_ and facilitates the conversion of less stable sulfate-containing phases into more stable carbonate-containing AFm phases [25]. In contrast, the 2.5%NC mixture exhibits a smaller reduction in portlandite intensity and a diminished calcite peak compared to the 2%NC mixture, indicating less efficient hydration and carbonate phase formation at the overdosage level. To move beyond purely qualitative visual inspection, a semi-quantitative analysis was performed based on the relative intensity ratios (RIR) of key crystalline phases. The integrated peak intensities of portlandite (Ca(OH)_2_, 2θ ≈ 18.1°), ettringite (AFt, 2θ ≈ 12.5°), AFm-CO_3_ (2θ ≈ 23.1°), and calcite (CaCO_3_, 2θ ≈ 29.4°) were normalized to the intensity of the internal quartz peak (2θ ≈ 26.6°), which served as a non-reactive internal standard. The results are summarized in Table 8.

The semi-quantitative data reveal two clear trends: (i) Portlandite consumption. The relative portlandite content in the 2%NC mixture is approximately 10% lower than that in the 0%NC reference (I/I_Q_ = 0.100 vs. 0.111), indicating enhanced consumption of Ca(OH)_2_ through nucleation-driven hydration and the formation of additional C-S-H gel. In the 2.5% NC mixture, the portlandite ratio returns to near the reference level (I/I_Q_=0.109), suggesting that excessive nano-CaCO_3_ leads to particle agglomeration, which hinders complete cement hydration. This observation is fully consistent with the UCS results, where the 2.5% mixture exhibited a statistically significant strength reduction. (ii) Carbonate phase evolution. The calcite peak intensity increases by approximately 8% in the 2%NC mixture compared to the 0%NC reference (I/I_Q_ = 0.719 vs. 0.663), reflecting both the direct contribution of unreacted nano-CaCO_3_ and the formation of carbonate-containing hydration products. Concurrently, the AFm-CO_3_ peak intensity decreases, suggesting a redistribution of carbonate species among different AFm phases. Notably, the 2.5%NC mixture exhibits a substantial decrease in calcite intensity (I/I_Q_ = 0.475), which is 28% lower than the reference value. This reduction is attributed to the poor dispersion and agglomeration of excess nanoparticles, which limits their effective participation in chemical reactions and reduces their detectability by X-ray diffraction.

The XRD analysis demonstrates that an optimal dosage of nano-CaCO_3_(2%) effectively accelerates cement hydration, promotes the consumption of portlandite, and enhances the formation of stable carbonate-containing phases. At an excessive dosage (2.5%), nanoparticle agglomeration impairs these beneficial effects, as evidenced by the reduced consumption of portlandite and the diminished calcite signal. These findings provide quantitative microstructural support for the observed mechanical behavior.

### 3.3. Scanning Electron Microscopy Test (SEM)

**Figure 7:**
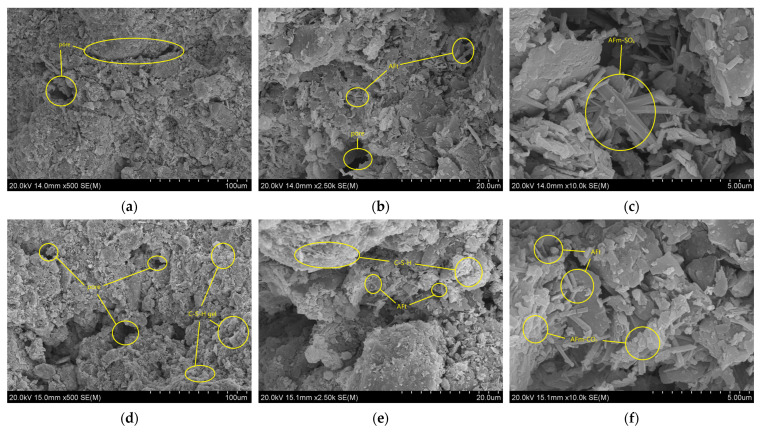
SEM Images of stabilized Soil: (**a**) cement stabilized (500×), (**b**) cement stabilized (2500×), (**c**) cement stabilized (10,000×), (**d**) composite stabilized (500×), (**e**) composite stabilized (2500×), (**f**) composite stabilized (10,000×).

The microstructural characteristics of cement-stabilized soil and 2% nano-calcium carbonate–cement composite-stabilized soil after 28 days of curing were examined using scanning electron microscopy (SEM). Representative images at magnifications of 500×, 5000×, and 10,000× are presented in Figure 7a–c and Figure 7d–f, respectively.

As shown in Figure 7a–c, the cement-stabilized soil exhibits pronounced macrocracks at 500× magnification, with limited hydration products observed between soil particles. The cementitious matrix appears insufficiently dense. Under external loading, the relative displacement of soil particles generates distinct cracks, accompanied by detachment of the interstitial cementitious materials—a common phenomenon in cement-based systems that contributes to primary crack formation and strength reduction [26]. At higher magnifications (2500× and 10,000×), minor quantities of C-S-H gel and ettringite are discernible. These hydration products partially fill interparticle voids and bind adjacent particles, leading to a moderate improvement in macroscopic mechanical properties. However, the overall limited hydration product content leaves a substantial proportion of soil particles loosely connected, restricting further strength gains.

In contrast, the composite-stabilized soil (Figure 7d–f) shows extensive coverage of soil particle surfaces by cement hydration products. Compared with Figure 7a, both the size and number of interparticle voids are significantly reduced, and interparticle bonding is markedly enhanced. At higher magnifications (Figure 7e–f), flocculent C-S-H gel becomes abundant on particle surfaces, while ettringite crystals appear finer, more numerous, and more densely distributed than in the cement-stabilized soil, often accumulating in layered stacks. These microstructural improvements are attributed to the nucleation effect of nano-calcium carbonate, which promotes the formation of uniform and dense C-S-H gel. Additionally, under the highly alkaline conditions of cement hydration, nano-calcium carbonate partially decomposes to release CO_3_^2−^ ions. These ions react with AFt and AFm-SO_4_ to form the more stable AFm-CO_3_, thereby inhibiting the conversion of AFt to AFm and mitigating void formation associated with volume shrinkage. This observation aligns with the findings of Niu W [27] regarding the influence of nano-calcium carbonate on ettringite in cement hydration products. To ensure the representativeness of the SEM observations, a minimum of five distinct regions per sample were examined at identical magnifications. The micrographs presented in Figure 7 are characteristic of the predominant microstructural features observed across these multiple scanned areas.

### 3.4. Particle (Pore) and Crack Analysis System (PCAS) Image Processing

To enable quantitative analysis of the micropore structure, the Particle (Pore) and Crack Image Recognition and Analysis System (PCAS) developed by Liu et al. [28] was employed.

Image Acquisition and Sampling Protocol. For each specimen type (cement-only and 2% NCC composite), five SEM micrographs were acquired at a consistent magnification of 500× from randomly selected, non-overlapping fields to ensure statistical representativeness. The fields were chosen to avoid image edges and obvious artifacts.

Binarization and Thresholding. The PCAS software (MatDEM5.00) automatically converts grayscale SEM images into binary (black/white) images. The binarization threshold was determined using the Otsu algorithm, which maximizes inter-class variance between pore pixels and solid matrix pixels. The same thresholding parameters were applied consistently across all analyzed images.

Reproducibility. The pore statistics reported in Table 9 represent the mean values calculated from the five independent image fields per specimen type, with standard deviations provided to indicate field-to-field variability.

**Figure 8:**
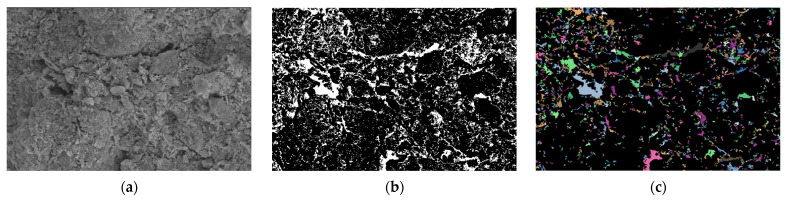
SEM image of cement-stabilized soil. (**a**) SEM image; (**b**) binarization; (**c**) vectorization.

**Figure 9:**
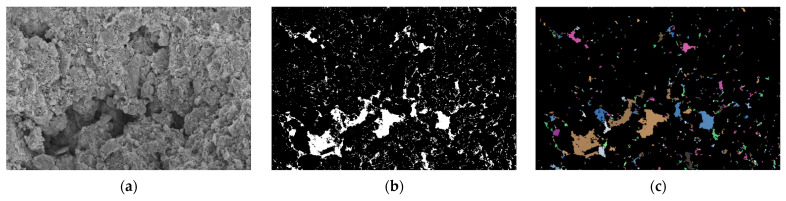
SEM image of Composite stabilized soil. (**a**) SEM image; (**b**) binarization; (**c**) vectorization.

Using PCAS, binarization was performed on the SEM images of both the cement-stabilized soil and the nano-calcium carbonate–cement composite-stabilized soil. Following binarization, the pore sizes and their spatial distributions became clearly discernible, as illustrated in Figure 8b and Figure 9b, White represents pores, and black represents the solid matrix.

Figure 8c and Figure 9c present the vectorized images of the SEM micrographs after binarization using the PCAS software. In these vectorized images, the colored irregular geometric shapes represent the stained pores. A comparison between Figure 8c and Figure 9c reveals that the cement-stabilized soil sample contains larger pores with an uneven distribution. In contrast, the soil sample treated with the cement–nano-calcium carbonate composite stabilizer exhibits a marked reduction in the number of large pores, while the small pores are more compactly and uniformly distributed. This indicates that the microstructure of the composite-stabilized soil differs significantly from that of the cement-only stabilized soil, with the former showing fewer large pores, an increased number of small pores, a more uniform pore size distribution, and a denser overall structure.

Table 9 presents the pore size distribution obtained from the SEM micrographs after vectorization using the PCAS software. It provides an intuitive and quantitative understanding of the number of small pores (D < 50 µm), medium pores (50 µm < D < 100 µm), and large pores (D > 100 µm), along with their respective surface pore area ratios.

Table 9 clearly presents the pore characteristics of the stabilized soils. The pore diameters are predominantly concentrated below 50 µm, with few pores exceeding 50 µm; the pore distribution is dominated by small and medium-sized pores. For pores with diameters D < 50 µm, the cement-stabilized soil contains 656 pores with a surface pore area ratio of 6.571%, whereas the cement–nano-calcium carbonate composite-stabilized soil contains 253 pores with a surface pore area ratio of 3.609%. This corresponds to reductions of 61.43% in pore count and 2.962% in surface pore area ratio. For pores in the range of 50 µm < D < 100 µm, the cement-stabilized soil exhibits 515 pores with a surface pore area ratio of 5.171%, while the composite-stabilized soil shows 148 pores with a surface pore area ratio of 2.145%, representing decreases of 71.26% and 3.026%, respectively. For pores with D > 100 µm, the cement-stabilized soil contains 389 pores with a surface pore area ratio of 3.898%, compared with 158 pores and a ratio of 2.293% in the composite-stabilized soil, corresponding to reductions of 59.38% and 1.605%, respectively.

The above charts and data indicate that, compared with cement-only stabilization, the microstructure of the soil treated with the composite stabilizer undergoes a notable transformation, characterized by a significant reduction in both pore count and porosity. This suggests that the soil particles are more effectively bonded, which facilitates the formation of stress-transfer pathways and enhances the capacity of the soil to function as a load-bearing framework.

### 3.5. Analysis of the Composite Solidification Mechanism

Based on the analysis of the experimental results, the solidification mechanism of dredged sludge treated with nano-calcium carbonate and cement can be attributed to three primary effects: nucleation, filling, and chemical reaction [29,30,31].

Owing to their small particle size, large specific surface area, and high surface energy, nano-calcium carbonate particles act as nucleation sites during cement hydration, providing additional sites for the formation of hydration products. The growth and coating of these products on the surfaces of nano-calcium carbonate particles accelerate the hydration reaction, leading to increased generation of C-S-H gel and enhanced early strength of the cement-stabilized soil. This is referred to as the nucleation effect.

Due to their finer particle size, nano-calcium carbonate particles can fill pores and microcracks within the cement-stabilized soil, thereby refining the pore structure. On one hand, they fill the large voids between soil particles, resulting in tighter interparticle contact; on the other hand, they penetrate the pores of cement hydration products, further reducing pore sizes and increasing the overall density, thereby improving the mechanical properties of the soil. This is known as the filling effect.

Cement contains substantial amounts of CaO, dicalcium silicate (C_2_S), tricalcium silicate (C_3_S), and tricalcium aluminate (C_3_A), which react with water to form Ca(OH)_2_, calcium silicate hydrate (C-S-H), and ettringite (AFt). In the sulfate-rich environment of dredged sludge, during the later stages of cement hydration, the remaining C_3_A reacts with previously formed AFt to convert into monosulfate-type AFm (AFm-SO_4_). However, with the incorporation of nano-calcium carbonate, the nano-calcium carbonate partially decomposes under the highly alkaline conditions of cement hydration, releasing CO_3_^2−^ ions. When AFm-SO_4_ (3CaO·Al_2_O_3_·CaSO_4_·12H_2_O) encounters CO_3_^2−^, the less stable SO_4_^2−^ is displaced from the interlayer, forming the more stable monocarbonate-type AFm (3CaO·Al_2_O_3_·CaCO_3_·11H_2_O). This process constitutes the chemical reaction effect.

[Ca_2_(Al,Fe)(OH)_6_]⋅SO_4_⋅12H_2_O + CO_3_^2−^→[Ca_2_(Al,Fe)(OH)_6_]⋅CO_3_⋅11H_2_O + SO_4_^2−^ + H_2_O, (1)

or simplified as:

AFm-SO_4_ + CO_3_^2−^→AFm-CO_3_ + SO_4_^2−^ + H_2_O (2)

The released CO_3_^2−^ ions not only react directly with AFm-SO_4_ but also inhibit the transformation of AFt (ettringite) into monosulfate-type AFm (AFm-SO_4_), thereby enhancing the stability of AFt. This is because the conversion of AFt to AFm-SO_4_ consumes SO_4_^2−^ and releases space; however, the competitive presence of CO_3_^2−^ favors the direct formation or conversion to the more stable AFm-CO_3_ phase. Furthermore, the formation of calcium aluminate carbonate consumes Ca^2+^ and OH^−^ from the system, reducing the alkalinity of the pore fluid to some extent. According to the principles of chemical equilibrium, this shift promotes further dissolution and hydration of cement clinker particles—particularly C_3_S—generating additional C-S-H gel, thereby establishing a self-reinforcing cycle that contributes positively to the overall hydration process.

## 4. Conclusions

This study systematically investigated the effects of nano-calcium carbonate as a supplementary admixture on the unconfined compressive strength and microstructural evolution of cement-stabilized dredged sludge. The main conclusions are as follows:

(1) Optimum dosage and strength enhancement. For a fixed cement content of 8% (by dry soil mass), the addition of 2% nano-CaCO_3_ (by cement mass) increased the 28-day UCS from 596 ± 18 kPa to 721 ± 15 kPa, representing a 21% improvement (*p* < 0.001). The enhancement was most pronounced at early ages (45% increase at 7 days), demonstrating the nucleation-driven acceleration of cement hydration.
(2) Detrimental effect of overdosage. Increasing the nano-CaCO_3_ content to 2.5% resulted in a statistically significant strength reduction (*p* = 0.003) relative to the 2% optimum. This is attributed to nanoparticle agglomeration, which creates localized weak zones and reduces the efficiency of hydration product formation, as evidenced by the smaller reduction in Portlandite XRD intensity at 2.5% compared to 2%.
(3) Microstructural mechanisms. The strength enhancement at optimal dosage arises from three synergistic mechanisms: (i) nucleation—providing additional surfaces for C-S-H precipitation; (ii) filling—refining the pore structure, as quantitatively confirmed by PCAS analysis showing a 61–71% reduction in pore counts across all size ranges; and (iii) chemical reaction—partial conversion of less stable AFm-SO_4_ to stable AFm-CO_3_ phases, identified through semi-quantitative XRD analysis.
(4) Practical implications and future work. The results confirm that nano-CaCO_3_ can serve as an effective, low-dosage additive to enhance the performance of cement-stabilized dredged sludge without increasing the cement content. Future research should focus on the long-term durability of the composite-stabilized material under wet–dry cycles, freeze–thaw conditions, and cyclic loading, as well as systematic substitution experiments to compare the effects of cement replacement versus additive approaches.

## Figures and Tables

**Table 1:** Basic Physical Parameters of Dredged Sludge.

Natural Water Content/%	Liquid Limit/%	Plastic Limit/%	Liquidity Index	Plasticity Index	Optimum Water Content/%	Maximum Dry Density/g/cm^3^
96	63.5	30.2	2.88	33.3	35	1.09

**Table 2:** Main Chemical Components of Dredged Sludge.

CaO	SiO_2_	Fe_2_O_3_	Al_2_O_3_	MgO	K_2_O	SO_3_	Na_2_O	TiO_2_	LOI
1.08%	59.44%	5.77%	27.88%	0.78%	2.33%	0.76%	0.28%	0.89	0.04

**Table 3:** Main Chemical Components of Portland cement.

CaO	SiO_2_	Fe_2_O_3_	Al_2_O_3_	MgO	K_2_O	SO_3_
59.27%	23.15%	3.23%	6.13%	2.24%	0.87%	3.91%

**Table 4:** Specifications for Nano-Calcium Carbonate.

Particle Size/nm	Specific Surface Area/(m^2^/g)	Bulk Density/(g/cm^3^)	CaCO_3_ Content/%	Density/(g/cm^3^)	Crystal Form	pH
10~20	150 ± 30	0.1–0.2	>99.9	2.44	γ	10

**Table 5:** Test Scheme.

Test Group	Cement Content (%)	Nano-Calcium Carbonate Content (%)	Age (Days)
Control group	0	0	7, 14, 28
Cement-cured group	8	0	7, 14, 28
Nano-calcium carbonate-cement-cured group	8	1, 1.5, 2, 2.5	7, 14, 28

**Table 6:** Comparison of Strength at Different Ages, Mix Proportions, and 28-Day Strength.

Age/d	Strength Ratio for Different Nano-Calcium Carbonate Content Levels				
	0%	1%	1.5%	2%	2.5%
7	0.49 ± 0.03	0.55 ± 0.02	0.55 ± 0.03	0.59 ± 0.02	0.47 ± 0.04
14	0.75 ± 0.03	0.74 ± 0.04	0.78 ± 0.03	0.83 ± 0.02	0.75 ± 0.03
28	1.00 ± 0.00	1.00 ± 0.00	1.00 ± 0.00	1.00 ± 0.00	1.00 ± 0.00

Note: The following analysis focuses on the 1.0% to 2.0% NCC content range, where a clear positive trend in strength development is evident.

**Table 7:** Summary of ANOVA and Tukey’s HSD Results for 28-day UCS.

Comparison	Mean Difference (kPa)	*p*-Value	Significance
2% vs. 0%	+125	<0.001	Yes
2% vs. 1%	+78	0.002	Yes
2% vs. 1.5%	+43	0.041	Yes
2% vs. 2.5%	+96	<0.001	Yes
1.5% vs. 2.5%	+53	0.089	No

**Table 8:** Relative peak intensity ratios (I/I_Q_) of key crystalline phases normalized to the quartz (101) peak at 26.6°2θ.

Phase	2θ/(°)	Plain Sludge	0%NC	2%NC	2.5%NC
Ettringite(AFt)	12.5	0.047	0.172	0.154	0.166
Portlandite(CH)	18.1	0.050	0.111	0.100	0.109
AFm-CO_3_	23.1	0.065	0.134	0.094	0.102
Calcite	29.4	0.053	0.663	0.719	0.475

**Table 9:** Pore distribution (Mean ± SD, *n* = 5 image fields).

Age/d	Cement-Stabilized Soil		Composite Stabilized Soil	
	Number of Pores/Unit	Surface Pore Ratio/%	Number of Pores/Unit	Surface Pore Ratio/%
d < 50	656 ± 22	6.571 ± 0.21	253 ± 15	3.609 ± 0.14
50 < d < 100	515 ± 18	5.171 ± 0.18	148 ± 11	2.145 ± 0.09
d > 100	389 ± 14	3.898 ± 0.12	158 ± 12	2.293 ± 0.08

## Data Availability

The original contributions presented in this study are included in the article. Further inquiries can be directed to the corresponding author.
